# Efficacy of Low-Level Laser Therapy in Reducing Pain in the Initial Stages of Orthodontic Treatment

**DOI:** 10.1155/2022/3934900

**Published:** 2022-06-14

**Authors:** Manoel Heitor Brito, Cinthya Quagliato Nogueira, Paula Cotrin, Tiago Fialho, Renata Cristina Oliveira, Ricardo Gobbi Oliveira, Samira Salmeron, Fabrício Pinelli Valarelli, Karina Maria Salvatore Freitas, Rodrigo Hermont Cançado

**Affiliations:** Department of Orthodontics, Inga University Center, Maringá, Paraná, Brazil

## Abstract

**Purpose:**

There is no consensus about the mechanism and efficacy in alleviating pain of the lower-level laser therapy (LLLT) during orthodontic treatment. This study aimed to evaluate the LLLT effectiveness clinically in reducing pain caused by orthodontic movement that occurs in the early stages of treatment.

**Methods:**

The sample consisted of 54 patients in need of orthodontic treatment divided into two groups. A 28 experimental patients group (initial mean age: 26.84 years old) was undergone gallium-aluminum-arsenide infrared laser application on 12 points for each tooth immediately after the installation of the first alignment archwire, and a 26 patients control group (initial mean age: 29.13 years old) was undergone to no pain control intervention at all. Pain intensity was measured by using a visual analog scale, which was marked pain level (mm) reported in 06, 24, 48, and 72 hours. The perception of pain (beginning, peak, decline, and absence) was evaluated by filling up a questionnaire. To compare the intensity and perception of pain between groups, a nonparametric Mann–Whitney has been performed.

**Results:**

The experimental group showed levels (mm) at 6 (*p* < 0.001), 24 (*p*=0.004), and 48 hours (*p*=0.007) and perception of pain (hours) in the peak (*p*=0.026), decline (*p*=0.025), and absence (*p*=0.008) significantly lower compared to the group control.

**Conclusion:**

Low-level laser therapy is effective in reducing pain severity caused by orthodontic forces activation, and it promotes the analgesic action lasting effect during the most painful feeling time.

## 1. Introduction

Patients undergoing orthodontic treatment may experience significant levels of pain, a common condition in orthodontic fixed appliance therapy [1–3], even discouraging them from seeking or continuing treatment [4]. Pain and discomfort affect virtually all patients undergoing orthodontic treatment, perhaps much more intensely than most orthodontists would imagine and is often cited as one of the negative aspects arising from all orthodontic treatment [3, 5].

The use of nonsteroidal anti-inflammatory drugs (NSAIDs) is the preferred method by orthodontists to control pain caused by tooth movement [3, 6]. Prescribing these drugs significantly reduces pain after the installation of the rubber separator or first alignment archwire [6–10]; however, the use of nonsteroidal anti-inflammatory drugs during treatment is still controversial because of its potential for interference with tooth movement, as its mechanism of action promotes inhibition of prostaglandin synthesis, responsible for the bone resorption process [7, 11].

Achieving an effective pain management method without drug administration is a common goal of research in all areas of the health sciences [12, 13]. For this reason, the use of laser has been increasingly frequent in dentistry, bringing benefits to patients in various specialties, including orthodontics.

Lasers are classified according to the power of their radiation emission, which can be high, medium, and low intensities. Low-level lasers (LLL), also called cold, therapeutic, or soft-laser lasers, have a photochemical action of analgesia, anti-inflammatory, and tissue biostimulation [10, 14] such as where the energy output is low enough so as not to cause an increase in the temperature of the treated tissue above 36.5°C, i.e., normal body temperature [15].

Different parameters of various types of lasers, such as time, intensity, energy density, number of irradiated points, and others, have been proven to be effective [10, 16, 17] for various purposes. The efficacy of lower-level laser therapy (LLLT) in reducing pain levels has been studied, and it may be due to increased local circulation, reduction in the production of inflammatory factors, and the release of inflammatory neurotransmitter [4, 6, 14]. Some studies were unsuccessful in controlling the painful sensation from orthodontic mechanics with the use of LLLT [15, 17, 18]. Actually, there is no consensus in the literature about the mechanism and efficacy in alleviating pain of the LLLT.

Thus, this study aimed to clinically evaluate the effectiveness of low-level laser therapy in reducing pain caused by orthodontic movement in the early stages of treatment.

## 2. Materials and Methods

### 2.1. Materials

This study was approved by the Ethics Research Committee of the Ingá University Center (protocol no. 768.652).

Sample size calculation was based on an alpha of 5% and a beta of 20%, using a standard deviation of previous research [19], of 15.68 mm measured in the VAS score at 6 hours in the laser group and considering a minimum difference to be observed between the groups of 10 mm. The results showed that 21 subjects were needed.

The sample consisted of 54 individuals, selected according to the following inclusion criteria: need for nonextraction orthodontic treatment performed with the preadjusted fixed appliances, 0.022″ slot; installation of the 0.012″ thermoactivated nickel-titanium archwire as the beginning of the alignment and leveling phase (Flexy Niti Thermal, Orthometric, Marília, Brazil); no-use medication that interferes with the results; good oral and general health conditions, and signed informed consent, agreeing with the research procedures.

The patients were randomly divided into two groups: laser group (LG) and control group (CG). The LG was composed of 28 patients: 13 male and 15 female, mean initial age of 26.84 years (SD 13.83), who received low-lever laser irradiation right after the installation of fixed appliances. The CG with 26 patients, 11 male and 15 female, mean initial age 29.13 years (SD 13.39) received no intervention for pain control after the appliance installation.

The laser used was a gallium-aluminum-arsenide (GaAlAs) infrared laser with a wavelength of 808 nm and a cross-sectional beam diameter of 2 mm (Three Light Plus, Clean Line, Taubaté, Brazil) adjusted to 40 mW.

### 2.2. Methods

**Figure 1:**
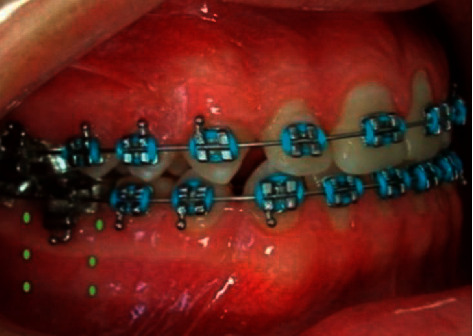
Points of laser irradiation in the buccal region of the right mandibular first molar.

Laser therapy was performed only once, immediately after brackets bonding and installation of the first archwire of the orthodontic treatment (0.012″ thermoactivated nickel-titanium). The average time for irradiation of all teeth was 19.5 minutes, on the distal region of the first molar to the distal region of the opposite first molar. Three points between the roots and distal spaces of the first molar (cervical, middle, and apical) of the buccal and lingual/palatal sides where the orthodontic appliance was installed (Figure 1) were irradiated for 15 seconds, 26 J/cm^2^ (0.78 J) per point, totaling 12 irradiations in each tooth, with a total energy of 9.36 J per tooth.

The level of pain was evaluated through a visual analog scale (VAS) with 100 mm horizontal line, delimited in three points with descriptors (expressions of smiles) in each marking: 0 (zero) meant no pain; 5 (five) representing moderate pain, and 10 (ten) representing severe pain. On this scale, the patient was instructed to mark a vertical risk at the location corresponding to the level of pain experienced at 06, 24, 48, and 72 hours after the orthodontic appliance installation.

The patients answered a questionnaire, with the VAS score at times evaluated, and indicated the perception of pain in relation to the amount of time from the laser therapy (initial), the highest intensity (maximum), and decline and absence of pain. Each patient indicated when (day and hour) these levels of pain was felt by them.

All research participants were told that it was not forbidden to take painkillers, but if they did, they should inform in the questionnaire which was the painkiller used, and these patients were excluded from the sample.

The compatibility between the groups for some characteristics that could influence the pain levels between the groups was also evaluated: age, Little' irregularity index to measure the crowding, sex, and type of malocclusion.

Little irregularity index measurements were obtained from the patients' dental casts by summing the linear displacements of the contact points between the incisors and between the lateral incisors and the mandibular canine [20]. Measurements of pain levels on the visual analog scale and Little's irregularity index were performed with a digital caliper (Mitutoyo America, Aurora III) with a measurement scale from 0 to 150 mm, a resolution of 0.01 mm. The type of malocclusion was evaluated in the initial dental casts.

## 3. Error Study

To assess the reliability of Little' irregularity index [20], new measurements were performed on 22 randomly selected dental casts after the one-month interval of the first measurement. The random errors were calculated according to Dahlberg's formula, and the systematic errors were evaluated with paired *t*-tests, at *p* < 0.05.

## 4. Statistical Analysis

Normal distribution of the variables was evaluated with Kolmogorov–Smirnov tests.

Intergroup comparability regarding ages and Little's irregularity Index at the initial stage was evaluated with *t*-tests. Intergroup comparability regarding sex distribution, malocclusion classification, and skeletal component was evaluated with chi-square tests. The level of pain symptomatology and pain perception between the two groups was evaluated with the Mann–Whitney test.

All statistical tests were performed with Statistica software (Statistica for Windows 10.0; Statsoft, Tulsa, USA). Results were considered statistically significant at *p* < 0.05.

## 5. Results

There was no systematic error, and the casual error was of 0.21 mm (Little' irregularity index).

The groups were comparable regarding initial ages, Little irregularity index, sex distribution, and type of malocclusion (Table 1).

**Figure 2:**
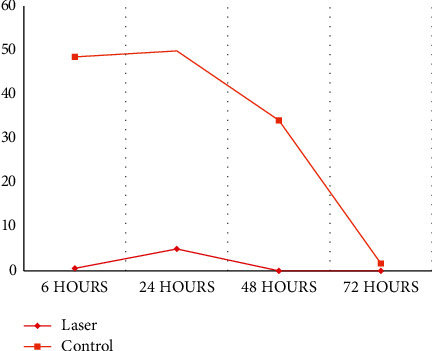
Level of painful symptoms in VAS score (mm) with time.

Patients that received laser therapy reported significantly lower levels of pain at 6, 24, and 48 hours than patients from the control group (Table 2). At the 72-hour evaluation, the pain level was almost absent and similar between the groups (Figure 2).

**Figure 3:**
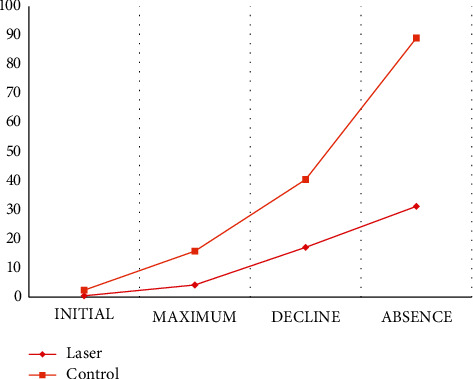
Elapsed time (hours) as a function of pain perception (initial, maximum, decline, and absence).

The laser group showed the maximum, decline, and absence of pain in significantly shorter periods of time than the control group (Table 3 and Figure 3).

## 6. Discussion

Both groups were comparable regarding initial ages, Little irregularity index, sex distribution, and type of malocclusion (Table 1). Therefore, the sample was homogeneous, and these variables did not influence the results. The lack of a placebo group was the major limitation of this study. The presence of a placebo group is essential when evaluating subjective outcomes, such as pain. However, it is possible to observe studies on the laser in the field of orthodontic pain carried out without a placebo group, just with a control group [21, 22].

The allocation of patients in each group was done consecutively and randomly, in a ratio of 1 : 1. The patients were sequentially distributed until each group reached the minimum amount required by the sample size calculation. One of the inclusion criteria was the no-use medication that interferes with the results, i.e., nonsteroidal anti-inflammatory drugs (NSAIDs) or painkillers. However, it was not prohibited for patients to take such medications if they felt it necessary. No patient took these medications, and there was no withdrawal.

The assessment of pain levels using the visual analog scale is a subjective method, but it is also one of the best available methods used to assess pain perception, used in several studies in the literature [1, 4, 6–8, 15, 17, 22–26]. VAS is a widely accepted, sensitive, and reliable method for measuring pain intensity [22].

The lower-level laser therapy (LLLT) irradiation protocol was based on the dosages used in the literature [21, 22]. A systematic review [27] concluded that laser therapy in the 810 nm range could be adjusted to significantly reduce inflammatory pain in clinical situations, with minimal doses of 6 Joules for small lesions and doses above 10 Joules for larger lesions (mean energy density of 7.5 J/cm^2^) administered within 72 hours of the injury. Another aspect considered was the time of 2-3 minutes per tooth, defined by Harazaki et al. [28] as necessary for the efficacy of LLLT in pain control. The gallium-aluminum-arsenide laser was chosen because of its greater tissue penetration depth, poor absorption by water molecules and macromolecules, and for being indicated for analgesia [29, 30]. A single dose of LLLT was applied in the present study and is in accordance with a research team from Pakistan [31, 32]. However, these same authors [33, 34] reported a reduction in pain associated with orthodontic treatment with low-level laser irradiation applied at 3-week intervals. A possible explanation for this different methodology is that it is also possible to obtain a greater rate of tooth movement associated with pain control with more applications.

The “Arndt-Schulz Law” is widely used to describe the effects of light parameters [35]. Energy density or dose, amount of energy per unit area transferred to matter, is an important parameter in LLLT, as it assesses the possibility of stimulation, inhibition, or nonmanifestation of the laser's therapeutic effects, but it is not restricted to it since different results are obtained when the same energy density is maintained and varying the power density and the exposure time [36, 37]. Some studies have used applied energy as if it was energy density [16, 38, 39] probably because the equipment sold commercially calculates energy density arbitrary for an area of 1 cm^2^ or by the cross-sectional area of the beam [37]. In fact, the irradiated density was much higher due to the exit area of the tips, which are small, between 0.03 and 0.04 cm^2^ in most devices.

In the present study, the laser was applied intraorally. However, transcutaneous (extraoral) use can also be necessitated in some cases due to the size of some kinds of laser device or operator preference. It is an essential question since the intra and extraoral administration of LLLT may have affected the results. The differences between the skin and mucosa affect the results, i.e., the effect of LLLT depends on the fact that laser light penetrates tissues and tissue fluids [30]. Aras and Güngörmüs [40] demonstrated that extraoral application is more effective than intraoral LLLT for the reduction of postoperative trismus and swelling after extraction of mandibular third molars. However, its use for pain reduction is still controversial [30]. Given this, the use of intraoral application for orthodontic pain relief seems to be more indicated since the application of the laser is made directly at the desired mucosa site, not having the skin barrier. Our results are in agreement with current studies [30–34].

The average time for irradiation of all teeth was 19.5 minutes, distal from molar to distal from opposite molar. Celebi et al. [22] completed the LLLT application per patient with an average duration of about 25 minutes. This time can be considered an unfavorable factor in the orthodontic routine since it requires a considerable chair time. However, we must take into account the fact that it is a noninvasive therapy [12, 41, 42] and has no side effects comparing to nonsteroidal anti-inflammatory drugs [6, 9, 43].

Therapy with nonsteroidal anti-inflammatory drugs is the method most used by orthodontists to control pain caused by fixed appliances [6]. However, these drugs inhibit the cyclooxygenase pathway and, as a consequence, decrease the production of prostaglandins that are responsible for osteoclastic activities and tooth movement [7], which generates controversies and fears of the routine use of these drugs during orthodontic treatment. However, some studies warn that due to the experimental work being done on animals that have a short life span, the doses of the medications are high and the administration periods are very long, reaching equivalent to 1/6 of the life of these animals [44] and that, therefore, it cannot be said about any change in tooth movement induced by some medication that the patient has used, since they are lower doses and for a short period of time after activation of orthodontic forces [6, 44].

The values of the levels of painful symptoms (Table 2 and Figure 2) indicate the effectiveness of laser irradiation after the installation of the orthodontic appliance. The experimental group had significantly lower pain levels in the periods of 06, 24, and 48 hours (*p* < 0.001, *p*=0.004, and *p* < 0.001), which is in line with previous research on the effectiveness of LLLT [16, 21, 23, 26, 27, 38, 39, 45, 46]. Although the pain level at 72 hours remained with lower values in the laser group, there was no statistically significant difference (*p*=0.235); however, the control group showed a quite intense decrease in pain levels in the interval between 48 (34.17; IR = 45.85) and 72 hours (1.71; IR = 32.67). According to the patients' perception of pain progression and decline (Table 3 and Figure 3), the control group showed a behavior similar to other studies [1, 4, 6, 23–26, 47]; however, the experimental group had shorter times during the period evaluated. The onset of pain was noticed in advance by the laser group, but there was no statistical significance, a finding that differs from other studies in which the painful sensation was perceived late [45, 46], but in agreement with the results of Tortamano et al. [39], where there was no statistically significant difference between the groups studied. The perception of decline and absence of pain in the experimental group (17.25 h and 31.23 h) and in the control group (40.59 h and 89.00 h) demonstrated that LLLT obtained a statistically significant efficacy (*p*=0.025 and *p*=0.008) of 97% and 99%, respectively. The patients showed improvement due to the anticipation of the attenuation and the absence of discomfort. Pain relief happened earlier and was even less intense during the comparison period between groups. If pain perception is examined only from the perspective of pain levels marked on the visual analog scale (Table 2 and Figure 2), it can be said that in both groups, the pain had similar behaviors: beginning at 6 h, peaking at 24 h, and there is a progressive decline in 48 and 72 hours.

The efficacy obtained with low-level laser therapy in the control of pain resulting from the activation of orthodontic forces in this study probably was a result of the parameters used is within the therapeutic window of low-level laser, mainly regarding the time of 3 minutes distributed in 12 points of 15 seconds, which provided laser penetration throughout each tooth.

It was found that the laser group had lower levels of painful symptoms throughout the study period, yet 72.22% of patients (laser group 60.71% and control group 84.61%) reported some level of pain in the first 24 hours, decreasing progressively in 48 and 72 hours (Table 2), while Scheurer et al. [1] observed reports of pain in 95% of their sample in the same time interval. This difference may be due to the 0.016″ nitinol archwire used in their work, whereas this study used the 0.012″ thermoactivated nickel-titanium archwire that has a lower elasticity module, more flexible, as it is of smaller caliber and the characteristic of maintaining practically the same force applied to the tooth after a certain tension, even suffering great deflections.

However, other studies with randomized clinical evaluation did not detect positive effects of laser therapy on reducing pain in the orthodontic treatment [19, 22]. There are conflicting results in the literature regarding the efficacy of LLLT for orthodontic pain [15, 21, 22, 48]. AlSayed Hasan et al. [19] evaluated laser therapy effects on reducing orthodontic pain in a randomized controlled trial, and the results from both groups found no effects in pain reduction. However, this contradiction may be due to the difference in the sample size of individuals in the experimental groups between studies and different energy laser irradiation values. Since different protocols and parameters were used, including application protocols and sample sizes, a variety of results was expected. Therefore, in this study, we tried to evaluate a larger experimental group comprised of 28 individuals. Studies using different sample sizes may lead to false conclusions. Thus, further studies with similar sample sizes should be carried out.

It is known that pain intensity is generally more prevalent in the inflammatory phase, during the first hours and days after the injury, and in most cases, the pain decreases as tissue repair processes occur [27]. To control the pain caused by the activation of the orthodontic appliance, pain killers need daily doses to maintain their effect, according to the time of their half-life, that is, they need to maintain effective therapeutic levels of these drugs in the body. Bernhardt et al. [7], Bradley et al. [8], and Steen Law et al. [9], when investigating the effectiveness of some NSAIDs, found that a percentage of patients used additional doses, as the doses administered in the experiment were not sufficient to maintain the analgesic action and control the pain caused by the orthodontic procedure. However, laser therapy demonstrated that only one irradiation provided statistically significant lower pain levels than the control group at 06, 24, and 48 hours. At 72 hours, there was no statistically significant difference, which can be attributed to the sharp decline in pain levels in the control group between 48 and 72 hours. Low-level laser therapy was able to provide a long-lasting analgesic action, with lower pain levels than the control group throughout the study period, which is in line with the results of Eslamian et al. [38] and Fujiyama et al. [26] who found statistically lower values during the first three and four days, that is, the effect of LLLT reduced the severity and/or the incidence of painful symptoms during the period of greater discomfort for patients. The separation period at the beginning of orthodontic treatment can also cause significant discomfort for the patient [49]. The maximum pain experienced after orthodontic elastomeric separation (OES) occurs on day 2 after the OES placement [50, 51]. Low-level laser therapy has been suggested as a method of controlling this pain [49, 51, 52]. Some studies compared the effect of LLLT and pain killers to reduce pain caused by elastomeric separators [51]. These studies showed that LLLT decreased the pain caused by elastomeric separators similarly to pain killers [51], whether it was applied as a single dose before separation or as a double dose before and after separation [49, 52].

Even though the pain levels were lower, a considerable portion of the sample experienced some discomfort during the initial phase of treatment. The forces applied to produce tooth movement can generate high levels of pain since it is subjective and related to each patient's experiences. It is up to the orthodontist to provide relief for the discomfort resulting from simple procedures such as installing intermaxillary elastics or leveling wires, as this undesirable effect can influence patients not to use important devices for therapy and even take them to abandon orthodontic treatment.

## 7. Conclusion

Low-level laser therapy showed to be effective in reducing pain severity in the early stages of orthodontic treatment, promoting long-lasting analgesic action during the period of greatest pain sensitivity.

## Figures and Tables

**Table 1:** Intergroup comparability.

Variables	Laser group (*n* = 28), mean (SD)	Control group (*n* = 26), mean (SD)	*P*
Age (years)	26.84 (13.83)	29.13 (13.39)	0.540^t^
Little irregularity index	4.94 (3.87)	5.19 (3.17)	0.794^t^
Gender			DF = 1
Male	13	11	*λ* ^2^ = 0.09
Female	15	15	0.760^x^
Type of malocclusion			
Class I	13	12	DF = 2
Class II	11	11	*λ* ^2^ = 0.10
Class III	04	03	0.947^x^

^t^Independent *t*-test. ^x^Chi-square test.

**Table 2:** Intergroup comparison of the levels of painful symptoms in VAS score (mm) in each time evaluated (nonparametric Mann–Whitney test).

VAS score (mm)	Laser group (*n* = 28)		Control group (*n* = 26)		*P*
	Median	IR	Median	IR	
6 hours	0.50	13.95	48.63	35.00	≤0.001^*∗*^
24 hours	5.04	33.31	50.00	48.14	0.004^*∗*^
48 hours	0.00	6.52	34.17	45.85	≤0.001^*∗*^
72 hours	0.00	2.86	1.71	32.67	0.235

^*∗*^Statistically significant difference (*p* < 0.05).

**Table 3:** Intergroup comparison of the elapsed time (hours) as a function of pain perception (initial, maximum, decline, and absence) (nonparametric Mann–Whitney test).

Pain perception (hours)	Laser group (*n* = 28)		Control group (*n* = 26)		*P*
	Median	IR	Median	IR	
Initial	0.50	3.53	2.50	3.00	0.066
Maximum	4.40	24.00	15.88	18.75	0.026^*∗*^
Decline	17.25	46.84	40.59	34.00	0.025^*∗*^
Absence	31.23	95.91	89.00	114.67	0.008^*∗*^

^*∗*^Statistically significant difference (*p* < 0.05).

## Data Availability

The VAS scores data used to support the findings of this study are available from the Uninga Institutional Review Board upon request (heitor75brito@gmail.com).
